# Genome Sequence of the Soviet/Russian *Bacillus anthracis* Vaccine Strain 55-VNIIVViM

**DOI:** 10.1128/genomeA.01401-16

**Published:** 2016-12-22

**Authors:** Jason Farlow, Adam Kotorashvili

**Affiliations:** a Farlow Scientific Consulting Company, LLC, Lewiston, Utah, USA; b Lugar Center for Public Health Research at National Center for Disease Control, Tbilisi, Georgia

## Abstract

*Bacillus anthracis* strain 55-VNIIVViM is a live-attenuated nonencapsulated Soviet/Russian veterinary anthrax vaccine strain. We report here the genome of 55-VNIIVViM and confirm its phylogenetic placement in the global population structure of *B. anthracis*.

## GENOME ANNOUNCEMENT

*Bacillus anthracis*, the causative agent of anthrax, is a Gram-positive, spore-forming bacteria that can cause acute infectious disease in livestock and wild ungulates (1). Humans are generally incidental hosts that acquire infection through handling contaminated meat or other animal products. Historically, *B. anthracis* was a subject of state-sponsored biowarfare research and currently poses a risk for illicit use as an agent of bioterrorism (2). *B. anthracis* exhibits global distribution and continues to present a significant public and animal health risk.

*B. anthracis* virulence is associated with two megaplasmids, pXO1 and pXO2 (3, 4). Toxigenic but attenuated live spore anthrax vaccines, lacking either of the virulence plasmids, have been in wide use since the 1930s. The most prevalent livestock vaccine is the live, nonencapsulated (pXO2) spore suspension of the Sterne strain developed in 1937 (5). In the Soviet Union, live-attenuated anthrax vaccine spore suspensions were also developed (6). Russian anthrax vaccine strains such as the Tsiankovskii-I strain and strain 55-VNIIVViM were developed for use in livestock. The Georgian/Soviet STI strain was also used as a livestock vaccine (6), in addition to its use in human vaccination, but was subsequently replaced by the 55-VNIIVViM strain in 1985. Currently, both commercial and national production facilities in the former Soviet Union maintain and distribute formulations of strain 55-VNIIVViM for regular use in veterinary vaccination programs. We report here the genome of *B. anthracis* strain 55-VNIIVViM.

A commercial preparation of strain 55-VNIIVViM was prepared for full-genome sequencing at the National Centers for Disease Control Lugar Center. Genomic DNA was extracted from colony culture using a Qiagen DNA mini prep kit, sheared to 350 bp. Whole-genome shotgun sequencing on the Illumina MiSeq platform produced 4,104,958 paired-end reads. Mapped and *de novo* read assemblies were analyzed using CLC Bio (http://www.clcbio.com) and Geneious version 7.0 (7) referenced against the Ames ancestor (NC_007530). *De novo* assembly yielded 31 chromosomal contigs and four contigs for the pXO1 plasmid. The 55-VNIIVViM draft genome was approximately 5,227,484 bp for the chromosome and 181,756 bp for the pXO1 plasmid with coverages of 188× and 481× for the chromosome and pXO1 plasmid, respectively. The RAST annotation server (8) and the NCBI Prokaryotic Genome Annotation Pipeline (9) were used for functional annotation using the Ames ancestor as a reference taxon.

Comparative sequence analyses identified at least 355 chromosomal polymorphisms that distinguish strain 55-VNIIVViM from the Ames ancestor. *In silico* canonical single-nucleotide polymorphism (canSNP) analysis placed 55-VNIIVViM within the trans-Eurasia group in the A.Br.008/009 lineage (10, 11). The B.Br.003 canSNP allele state in the strain 55-VNIIVViM genome reported here (B.Br.003=G) differs from that reported by Afanas’ev et al. (B.Br.003=A) (12). In addition, preliminary whole-genome comparisons suggest strain 55-VNIIViM falls within the newly identified STI group (13). The genome of strain 55-VNIIVViM has been made available for further cross-strain comparisons with other global representatives of *B. anthracis*.

### Accession number(s).

The whole-genome shotgun project for strain 55-VNIIVViM has been deposited at DDBJ/ENA/GenBank under the accession number MLJX00000000. The version described in this paper is the first version, MLJX01000000.

## References

[1] Turnbull PC 2002 Introduction: anthrax history, disease and ecology. Curr Top Microbiol Immunol 271:1–19. doi:10.1007/978-3-662-05767-4_1. 12224519

[2] Inglesby TV, Henderson DA, Bartlett JG, Ascher MS, Eitzen E, Friedlander AM, Hauer J, McDade J, Osterholm MT, O’Toole T, Parker G, Perl TM, Russell PK, Tonat K 1999 Anthrax as a biological weapon: medical and public health management. JAMA 281:1735–1745. doi:10.1001/jama.281.18.1735. 10328075

[3] Mock M, Fouet A 2001 Anthrax. Annu Rev Microbiol 55:647–671. doi:10.1146/annurev.micro.55.1.647. 11544370

[4] Leppla SH 1995 Anthrax toxins, p 543–572. *In* Moss J, Iglewski B, Vaughn M, Tu AT (ed.), Bacterial toxins and virulence factors in disease. Marcel Dekker, New York, NY.

[5] Sterne M 1939 The use of anthrax vaccines prepared from avirulent (uncapsulated) variants of *Bacillus anthracis*. Onderstepoort J Vet Sci Anim Ind 13:307–312.

[6] Shlyakhov EN, Rubinstein E 1994 Human live anthrax vaccine in the former USSR. Vaccine 12:727–730. 8091851 10.1016/0264-410x(94)90223-2

[7] Kearse M, Moir R, Wilson A, Stones-Havas S, Cheung M, Sturrock S, Buxton S, Cooper A, Markowitz S, Duran C, Thierer T, Ashton B, Meintjes P, Drummond A 2012 Geneious Basic: an integrated and extendable desktop software platform for the organization and analysis of sequence data. Bioinformatics 28:1647–1649. doi:10.1093/bioinformatics/bts199. 22543367 PMC3371832

[8] Aziz RK, Bartels D, Best AA, DeJongh M, Disz T, Edwards RA, Formsma K, Gerdes S, Glass EM, Kubal M, Meyer F, Olsen GJ, Olson R, Osterman AL, Overbeek RA, McNeil LK, Paarmann D, Paczian T, Parrello B, Pusch GD, Reich C, Stevens R, Vassieva O, Vonstein V, Wilke A, Zagnitko O 2008 The RAST server: rapid annotations using subsystems technology. BMC Genomics 9:75. doi:10.1186/1471-2164-9-75. 18261238 PMC2265698

[9] Angiuoli SV, Gussman A, Klimke W, Cochrane G, Field D, Garrity G, Kodira CD, Kyrpides N, Madupu R, Markowitz V, Tatusova T, Thomson N, White O 2008 Toward an online repository of standard operating procedures (SOPs) for (meta)genomic annotation. Omics 12:137–141. doi:10.1089/omi.2008.0017. 18416670 PMC3196215

[10] Van Ert MN, Easterday WR, Huynh LY, Okinaka RT, Hugh-Jones ME, Ravel J, Zanecki SR, Pearson T, Simonson TS, U’Ren JM, Kachur SM, Leadem-Dougherty RR, Rhoton SD, Zinser G, Farlow J, Coker PR, Smith KL, Wang B, Kenefic LJ, Fraser-Liggett CM, Wagner DM, Keim P 2007 Global genetic population structure of *Bacillus anthracis*. PLoS One 2:e461. doi:10.1371/journal.pone.0000461. 17520020 PMC1866244

[11] Kenefic LJ, Pearson T, Okinaka RT, Schupp JM, Wagner DM, Hoffmaster AR, Trim CB, Trim CP, Chung WK, Beaudry JA, Jiang L, Gajer P, Foster JT, Mead JI, Ravel J, Keim P 2009 Pre-Columbian origins for North American anthrax. PLoS One 4:e4813. doi:10.1371/journal.pone.0004813. 19283072 PMC2653229

[12] Afanas’ev MV, Kravets EV, Dugarzhapova ZF, Takaishvili VE, Polovinkina VS, Balakhonov SV 2014 Comparative multilocus VNTR and SNP analysis of *Bacillus anthracis* vaccine strains. Mol Genet Microbiol Virol 29:86–92. doi:10.3103/S0891416814020025. 25080817

[13] Sahl JW, Pearson T, Okinaka R, Schupp JM, Gillece JD, Heaton H, Birdsell D, Hepp C, Fofanov V, Noseda R, Fasanella A, Hoffmaster A, Wagner DM, Keim P 2016 A Bacillus anthracis genome sequence from the Sverdlovsk 1979 autopsy specimens. mBio 7:e01501-16. 27677796 10.1128/mBio.01501-16 PMC5050339

